# A new approach to Cas9-based genome editing in *Aspergillus niger* that is precise, efficient and selectable

**DOI:** 10.1371/journal.pone.0210243

**Published:** 2019-01-17

**Authors:** Laure M. C. Leynaud-Kieffer, Samuel C. Curran, Irene Kim, Jon K. Magnuson, John M. Gladden, Scott E. Baker, Blake A. Simmons

**Affiliations:** 1 Swiss Federal Institute of Technology Lausanne, Lausanne, Vaud, Switzerland; 2 Joint Bioenergy Institute, Emeryville, CA, United States of America; 3 Biological Systems and Engineering Division, Lawrence Berkeley National Laboratory, Berkeley, CA, United States of America; 4 Comparative Biochemistry Graduate Group, University of California Berkeley, Berkeley, CA, United States of America; 5 Department of Chemistry, University of California, Berkeley, CA, United States of America; 6 Chemical and Biological Process Development Group, Pacific Northwest National Laboratory, Richland, WA, United States of America; 7 Department of Biomass Science and Conversion Technology, Sandia National Laboratories, Livermore, CA, United States of America; 8 Biosystems Design and Simulation Group, Environmental Molecular Sciences Division, Pacific Northwest National Laboratory, Richland, WA, United States of America; Woosuk University, REPUBLIC OF KOREA

## Abstract

*Aspergillus niger* and other filamentous fungi are widely used in industry, but efficient genetic engineering of these hosts remains nascent. For example, while molecular genetic tools have been developed, including CRISPR/Cas9, facile genome engineering of *A*. *niger* remains challenging. To address these challenges, we have developed a simple Cas9-based gene targeting method that provides selectable, iterative, and ultimately marker-free generation of genomic deletions and insertions. This method leverages locus-specific “pop-out” recombination to suppress off-target integrations. We demonstrated the effectiveness of this method by targeting the phenotypic marker *albA* and validated it by targeting the *glaA* and *mstC* loci. After two selection steps, we observed 100% gene editing efficiency across all three loci. This method greatly reduces the effort required to engineer the *A*. *niger* genome and overcomes low Cas9 transformations efficiency by eliminating the need for extensive screening. This method represents a significant addition to the *A*. *niger* genome engineering toolbox and could be adapted for use in other organisms. It is expected that this method will impact several areas of industrial biotechnology, such as the development of new strains for the secretion of heterologous enzymes and the discovery and optimization of metabolic pathways.

## Introduction

The recombinant production of enzymes at high titers using various hosts, such as filamentous fungi, is an important aspect affecting costs for many commercial applications today, including pharmaceuticals [1], food processing [2], biofuels [3], and detergents. Despite the widespread deployment of these fungal strains in industry, the genetic toolbox by which they can be efficiently optimized for any given application, such as improved recombinant protein production from gene expression, remains challenging and time consuming [4]. One of the industrial approaches to the conversion of starches and polysaccharides into monomers suitable for subsequent bioconversion into biofuels relies on the use of hydrolytic enzymes, such as amylases, cellulases, and hemicellulases that are naturally found in fungi and bacteria [5,6]. In order for recombinant enzymes of this type to be produced at the commercial scale, they must be produced at high titers and yields in order to reduce costs. While these enzymes could be produced by the filamentous fungi in which they are found in naturally or in recombinant hosts, these fungi may not secrete enough of the targeted enzymes needed and therefore genetic engineering and optimization of these strains is an important component of commercial viability [7].

*Aspergillus niger* is a filamentous ascomycete fungus utilized industrially for the production of citric acid and for its ability to produce and secrete high levels of endogenous and recombinant enzymes [8]. It is generally recognized as safe at the commercial scale, its genome is sequenced and it is amenable to standard genetic modification techniques [9]. The genomic integration of exogenous DNA via homologous recombination (HR) has been widely applied in *A*. *niger* and other filamentous fungi [8]. Typically, genes are replaced with a “fixing template” containing a selectable marker, thereby permitting selection of the integration event. The *pyrG* gene, encoding encodes orotidine-5′-monophosphate decarboxylase, an intermediate in the pyrimidine pathway forming uridine monophosphate, is both positively and negatively selectable; the integration of *pyrG* can be selected for by culturing in the absence of uracil/uridine while the absence of *pyrG* can be selected for in the presence of 5-fluoroorotic acid (5-FOA) [10,11]. *pyrG* converts 5-FOA into fluoroorotidine monophosphate which is subsequently converted into fluorodeoxyuridine by ribonuclease reductase. Fluorodeoxyuridine is a suicide inhibitor of the thymidylate synthase and therefore inhibits DNA synthesis and leads to cell death. 5-FOA is non-toxic in the absence of *pyrG*. The positive/negative selection of *pyrG* can be exploited to permit iterative targeting by selecting for the “pop-out” excision of *pyrG* via HR after integration [12].

Targeting double stranded breaks (DSBs) to the site of DNA integration is known to increase the efficiency of HR [13–16]. Originally a bacterial defense system, the now-ubiquitous CRISPR/Cas9 (Clustered Regularly Interspaced Short Palindromic Repeats; CRISPR associated protein 9) was engineered for rapid targeting of DSBs [17]. In this system, a small guide RNA (sgRNA) targets the Cas9 endonuclease to its complementary DNA. In addition to facilitating HR, CRISPR/Cas9 can be used to introduce deletions and point mutations without necessarily introducing foreign DNA [18,19]. CRISPR/Cas9 was previously demonstrated to be effective in several filamentous fungi, e.g. *A*. *niger*, *A*. *oryzae*, *A*. *fumigatus*, and *Neurospora crassa* [20–22].

Nevertheless, this method requires extensive screening as off-target integrations, mediated by non-homologous end-joining (NHEJ), lead to an overwhelming rate of false positives [21]. Several strategies have been employed to increase the efficiency of HR, including the adjustment of length of the HR arms [23], engineering the RAD52 HR protein [24], or knocking out the *Ku70* genes responsible for NHEJ [25]. Complete disruption of NHEJ can lead to genomic instability and increases the risk of DNA damage [26]. Therefore, high-efficiency specific gene editing in *A*. *niger* and other filamentous fungi remains a significant challenge. Editing efficiency has been reported to be from anywhere between 1 and 100% efficient depending on the CRISPR/Cas9 setup and the target locus [21]. Targeting non-phenotypic genes requires laborious sequencing of transformants.

To address these challenges, we have developed reusable, transiently-selectable donor DNA for a specific integration system. After validating this methodology using the phenotypic marker *albA*, we sequentially targeted two genes likely to improve heterologous enzyme production. We replaced *glaA* (glucoamylase) with the *Thermotoga petrophila* β-glucosidase designated A5IL97 [27]. We then interrupted the sugar transporter *mstC* [28] and observed 100% efficiency of the desired mutations at all three loci using positive and negative selection pressure. This approach allows for the efficient engineering of *A*. *niger* and eliminates the need for screening hundreds of transformants. To the best of our knowledge, this is the first published report on this new Cas9 approach and applying it in *A*. *niger* (or any fungi) and significantly reduces the time required for the screening of positive mutants at high efficiencies.

## Results

Our approach relies on the induction of a genomic DSB with a targetable Cas9/sgRNA complex, incorporation of a selectable marker via HR, and selection of *pyrG*-containing mutants by culturing in the absence of uracil/uridine. To validate this approach, we targeted *albA*, a polyketide synthase responsible for the production of a black spore pigment [29]. When *albA* is disrupted, colonies present a white rather than black spore phenotype, providing a convenient and commonly used selection technique.

**Fig 1:**
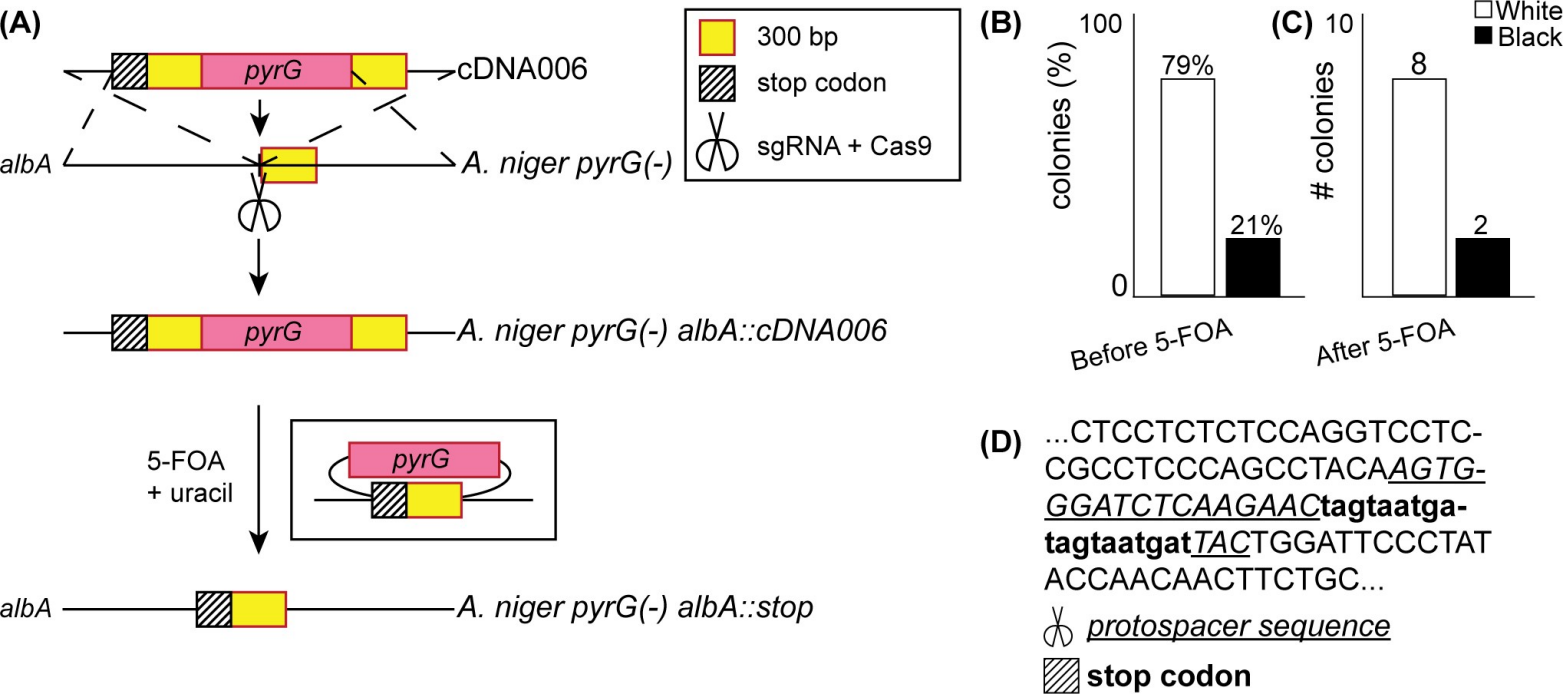
Design and application of cDNA006 for disruption of *albA*. (A) of cDNA006 contains *pyrG* flanked by 300bp repeats at 1000 bp homology arms to *albA*. After integration, *pyrG* is excised by homologous recombination in the presence of 5-FOA. (B) and (C) Phenotypes obtained before 5-FOA and after 5-FOA. (D) Representative sequence showing the integration of the stop codon at the *albA* locus in a white colony.

We generated a fixing template cDNA006, with 1,500 bp homology arms for targeting *albA* (Fig 1). cDNA006 contains a 5’ stop codon repeat for disrupting translation and the *pyrG* gene. To generate a “recyclable” marker system, *pyrG* was flanked with direct repeat sequences [12]. Upon exposure to 5-FOA, transformants containing *pyrG* should undergo “pop-out” recombination to remove the marker, thereby permitting additional rounds of gene targeting using *pyrG* selection.

While some methods contain the fixing template and sgRNA on the same plasmid as Cas9, this necessitates additional cloning steps when targeting new genes and leads to off target effects due to constitutive expression [21]. We therefore opted for *in vitro* preparation of the sgRNA and fixing template (see Methods). cDNA006, an *albA*-targeting small guide RNA (sgRNA001) and plasmid pFC332, containing a constitutively expressed *A*. *niger* codon-optimized Cas9, were simultaneously transformed into ATCC 1015 *pyrG* ^-^. Transformants were plated onto minimal media without uracil/uridine and with 300 μg/mL hygromycin to select for the integration of *pyrG* and the maintenance of pFC332, respectively. After 4 days, 79% of the colonies had the white spore phenotype, indicating successful targeting of *albA* (Fig 1). We then isolated black and white colonies and re-streaked them on minimal media containing uracil/uridine and 5-FOA, to select for the “pop-out” recombination of *pyrG* (Fig 1A, step 2). These colonies were then re-plated on MMA + uracil. Sequencing the specific locus revealed that the 100% of the black colonies were free of mutations at the *albA* locus, while 100% of the white colonies contained the integrated stop codon exact protospacer location of the sgRNA (Fig 1) (S1 Fig).

We observed efficient, selectable gene deletion with successful excision of *pyrG*. Nevertheless, 21% of colonies did not have mutations at the *albA* locus but survived on MMA + hygromycin without uracil/uridine supplied (Fig 1B and 1C), indicating NHEJ-mediated off-target integration of the fixing template [30]. While NHEJ-mediated repair can be suppressed by knocking out genes in the NHEJ pathway, this can lead to genomic instability and mutagenic sensitivity [26]. Therefore, we sought to engineer a fixing template to screen positive mutations at the correct integration locus.

### Developing a specific pop-out marker

**Fig 2:**
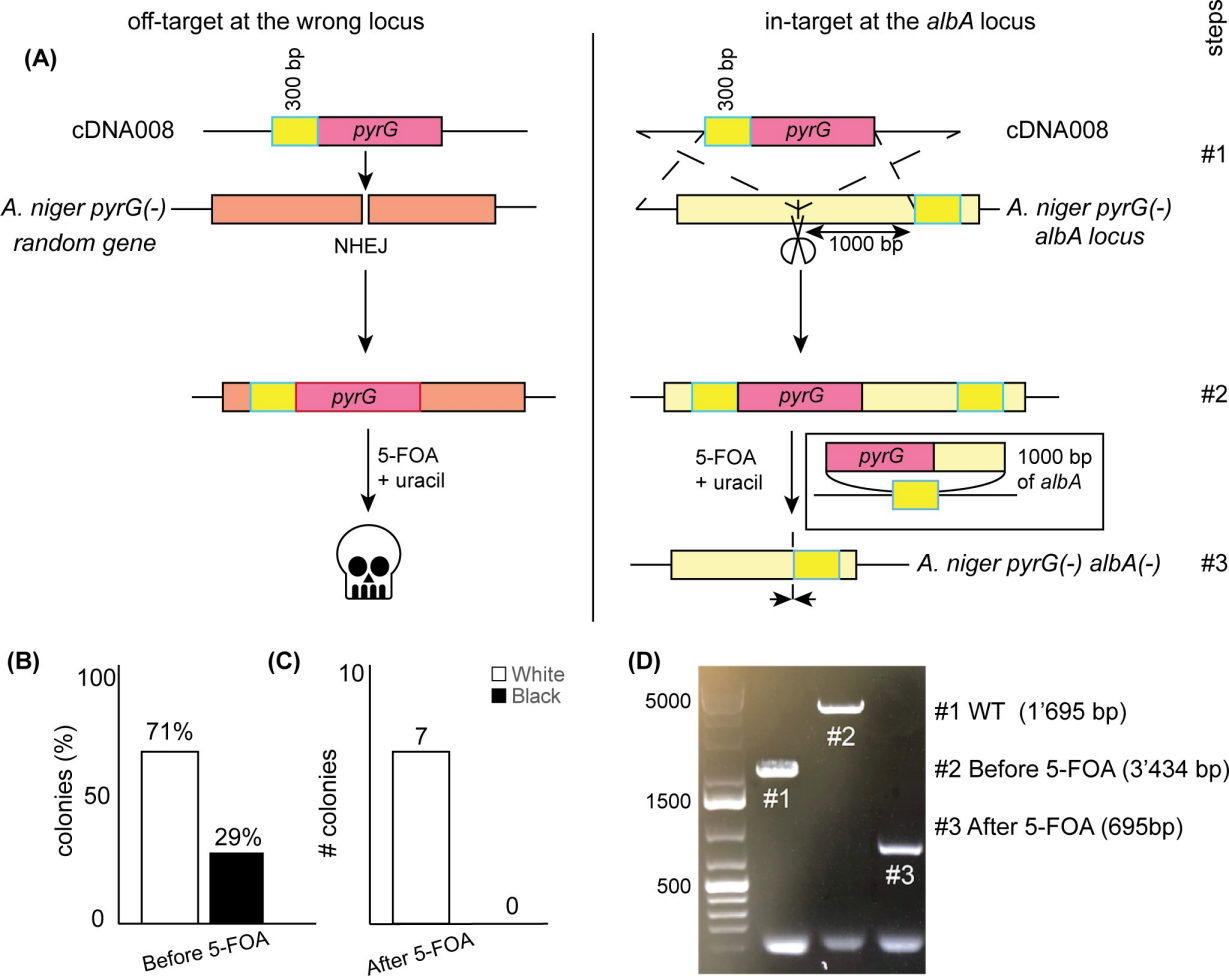
Design and application of a specific construct cDNA008 for disruption of *albA*. (A) Design of cDNA008 construct inserted at the *albA* locus to delete 1,000 bp making *A*. *niger pyrG−albA*^*–*^, use of sgRNA001. (B) and (C) Results obtained before 5-FOA and after 5-FOA. (D). PCR amplification of the *albA* gene in wild type (WT) strain #1, before 5-FOA insertion of *pyrG* at the *albA* locus #2, and deletion of 1000 bp of *albA* after 5-FOA #3.

We designed a fixing template (cDNA008) that will excise *pyrG* when it is specifically integrated at the *albA* locus (Fig 2A). Rather than inserting a stop codon, cDNA008 was designed to delete 1000 bp of *albA* to disrupt the gene. Like cDNA006, cDNA008 contains the *pyrG* gene. A 300 bp cassette was placed in front of the *pyrG* gene that are homologous to the 3’ region of *albA*. After integration and exposure to 5-FOA, *pyrG* should undergo pop out recombination if it is correctly integrated into the *albA* locus. HR loses efficiency as the distance between homologous sequences increases [31]. Therefore, HR-mediated excision of *pyrG* will be inefficient for off-target integrations, and cells with off-target integrations should die in the presence of 5-FOA.

After transformation of Cas9, sgRNA001, and cDNA008, 71% of the colonies had the white spore phenotype (Fig 2B). 7 white and 3 black colonies were re-streaked on plates containing 5-FOA. The white colonies survived on plates containing 5-FOA, while there was no detectable growth of the black colonies after one week (Fig 2C) (S2 Fig). PCR amplification of the *albA* locus at each stage showed (#2) the integration of *pyrG*, and (#3) the pop-out recombination of *pyrG* and deletion of 1000bp of *albA* (Fig 2D). Sequencing the *albA* locus of all mutants confirmed the integration of *pyrG* and subsequent recombination upon 5-FOA treatment. Therefore, on the 10 analyzed colonies, we observed 100% of correct *albA* locus modifications after treatment with 5-FOA, suggesting the method suppresses off-target integrations (S2 and S3 Figs).

### Targeting a non-phenotypic gene

**Fig 3:**
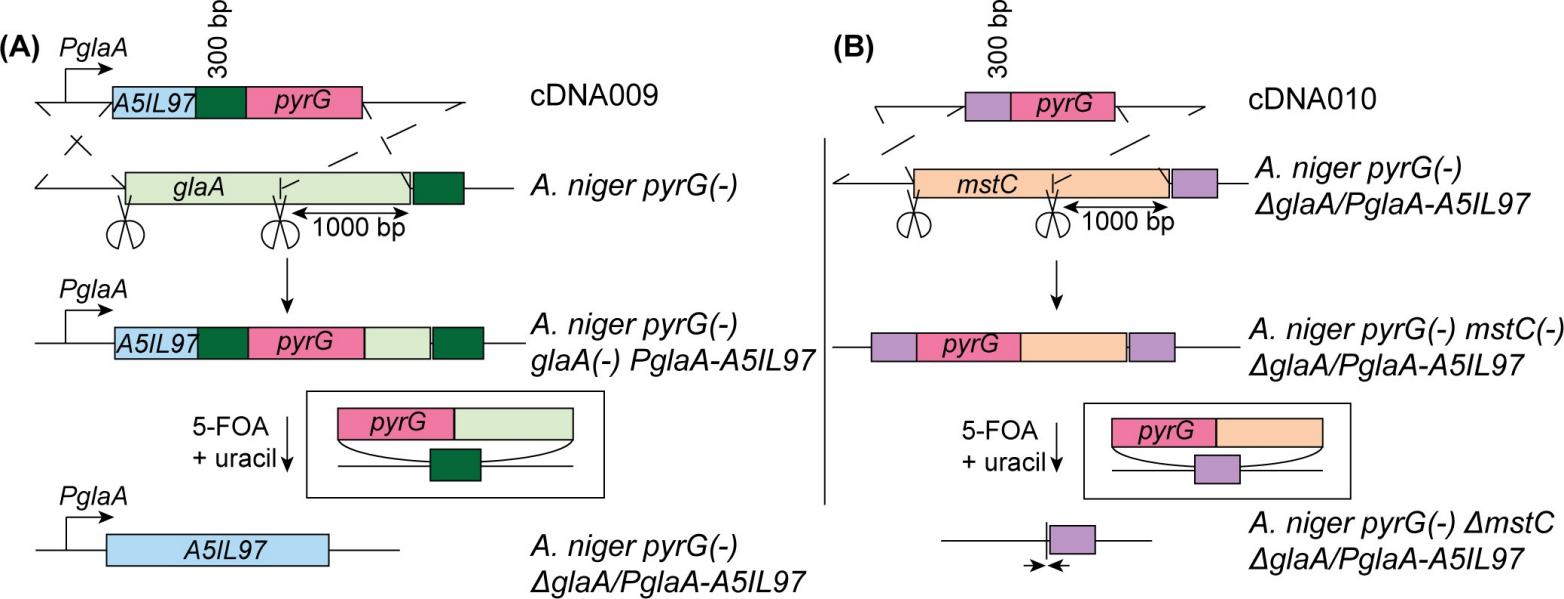
Design and application of cDNA009 (*glaA* locus) and cDNA010 (*mstC* locus). (A) cDNA009 construct inserted at the *glaA* locus to insert *A5IL97* gene making *A*. *niger pyrG− ΔglaA/P*_*glaA*_*-A5IL97*, use of sgRNA002 and sgRNA003. (B) Design of cDNA010 construct inserted at the *mstC* locus to delete *mstC* on the *A*. *niger pyrG− ΔglaA/P*_*glaA*_*-A5IL97*, resulting in the strain *A*. *niger pyrG− ΔglaA/P*_*glaA*_*-A5IL97 ΔmstC*.

After demonstrating the feasibility of our method at the *albA* locus, we then targeted the non-phenotypic gene *glaA*, and replaced it with another gene, *A5IL97*, in a single procedure. The *glaA* gene encodes the glucoamylase enzyme, a natural highly secreted enzyme of *A*. *niger* [32], which has a strong promoter, *P*_*glaA*_ [33], that can be used to produce heterologous enzymes [28]. As a proof of concept, we used the gene that encodes for the β-glucosidase *A5IL97* that has been previously shown to be secreted by *A*. *niger* [28]. We designed a construct, cDNA009, to target the *glaA* locus. cDNA009 resembles the cDNA008 with the addition of the open reading frame (ORF) for A5IL97 (Fig 3A). After transformation, 10 colonies were isolated on MMA selecting for the integration of *pyrG*. After PCR amplification at the *glaA* locus, only 8 colonies of the 10 selected on MMA had integration of the *pyrG* marker at the locus. After 5-FOA selection, only the 8 colonies containing previously *pyrG* survived on 5-FOA. Sequencing of 5-FOA resistant mutants confirmed 100% efficient deletion of *glaA*, integration of A5IL97 and the *pyrG* marker was removed at the locus (Table 1) (S4 Fig).

**Table 1:** Efficiency obtained before and after selection of 5-FOA by PCR amplification at the mutated locus and sequence verified.

Gene targeting	Constructs	sgRNA	Method	Before 5-FOA	After 5-FOA
***albA−*** Codon stop insertion	cDNA006 X-pyrG-X	sgRNA001	Non-selective	19 white colonies 5 black colonies (79% white colonies)	8 white colonies 2 black colonies
***albA−*** Deletion of 1000 bp	cDNA008 X-pyrG	sgRNA001	Selective	20 white colonies 8 black colonies (71% white colonies)	7 white colonies 0 black colonies
***ΔglaA*** Gene replacement with A5IL97	cDNA009 A5IL97-X-pyrG	sgRNA002 sgRNA003	Selective	8 colonies with *pyrG* at the locus 2 colonies without *pyrG* at the locus	8 on target 0 off target
***ΔmstC*** Gene deletion	cDNA010 X-pyrG	sgRNA004 sgRNA005	Selective	7 colonies with *pyrG* at the locus 3 colonies without *pyrG* at the locus	7 on target 0 off target

As 5-FOA exposure led to the excision of *pyrG* and the genotype *A*. *niger ΔglaA/P*_*glaA*_*-A5IL97 pyrG−*, this method is inherently recyclable. After successfully replacing *glaA* with A5IL97, we verified the iterative nature of this method by targeted disruption of a second gene, *mstC*, in this strain (Fig 3B). *mstC* encodes a glucose transporter that, once disrupted, has been identified to enhance the *P*_*glaA*_ for heterologous enzyme production [28]. With an off-target suppressing construct, we targeted *mstC* and observed 100% deletion after 5-FOA (Table 1), making the strain *A*. *niger pyrG− ΔglaA/P*_*glaA*_*-A5IL97 ΔmstC* (S5 Fig).

## Discussion

We have designed and demonstrated a technique that efficiently edits the genome of *A*. *niger* based on CRISPR/Cas9. We targeted the non-phenotypic genes *glaA* and *mstC* on the same strain and obtained 100% efficiency after selection on 5-FOA. Despite the 100% efficiency observed at these three different loci using the method, there is no guarantee that 100% efficiency will be observed for all loci. Many factors influence the probability of genomic modification, including the essentiality and accessibility of a gene [34]. The originality of this technique is in the design of the construct which leads to a simple counter selectable method for in-target integration, allowing us to tolerate loss of efficiency due to the organism, the gene target [35], the choice of the sgRNA or the way in which it is delivered (*in vitro* or *in vivo*, choice of the promoter), and the Cas9 expression method. It should be noted that other off-target effects, such as the generation of point mutants caused by Cas9, are not suppressed. The method presented here should overcome limitations in genome editing in filamentous fungi such as low efficiency editing for some loci and the time required to screen mutants when the gene in target is not phenotypic. The described method is a worthwhile addition to the tools available for genome editing in filamentous fungi such as the use of short recombination arms [36], and reduction of off-target effects by knockout of the NHEJ protein *KusA* [37].

We used the Cas9 plasmid under a constitutive promoter but not with the sgRNA on the plasmid to reduce the risk of off-target effects [19,38] and facilitate the preparation of the sgRNA for the transformation. For our purposes *in vitro* sgRNA preparation was sufficient for 100% gene editing, which is in line with other reports demonstrating the efficiency of *in vitro* sgRNA [30,39]. The choice of the sgRNA is crucial for the Cas9 targeting efficiency. A simple test *in vitro* with Cas9 can demonstrate the efficiency of each individual sgRNA (see Methods). Looking forward, *in vitro* sgRNA preparation may be the easiest method for testing many sgRNAs without the need for extensive sub cloning [30].

The primary focus of this study was to reduce the workload of screening for positive mutants and to generate a recyclable rescue marker for iterative mutation, which we have demonstrated. This method can be adopted to generate point mutants by incorporating the mutation in the fixing template. In this study we only used the auxotrophic marker *pyrG* vs 5-FOA, but there are more rescue markers available that have not been tested, such as *amdS*. This method may be applied to multiplex genome engineering in the same recyclable, specific manner. Many of the pre-existing CRISPR/Cas9 methods work in multiple filamentous fungi [21]. While we have only tested these methods on *A*. *niger*, these methods may likely be applied to other species. In conclusion, this novel method greatly simplifies genome editing in *A*. *niger* and will enable the rapid generation of genomic mutants and libraries for the investigation of biology and further improve the use of *A*. *niger* as an important heterologous production host.

## Materials and methods

### Reagents

All chemicals were purchased from Sigma unless otherwise noted.

### Strains

The strains used in this paper are listed in Table 2. The genome sequence of strain ATCC 1015 v4.0 is accessible from the Joint Genome Institute (JGI).

**Table 2:** *A*. *niger* strains used in this study and their accession information.

Name	Genotype	Source	Access
JBEI-14377	*ATCC 1015 pyrG* ^*-*^	[29]	https://registry.jbei.org/folders/1399
JBEI-099147	*ATCC 1015 pyrG−albA –*	This study.	https://registry.jbei.org/folders/1399
JBEI-099148	*ATCC 1015 pyrG−albA –*	This study.	https://registry.jbei.org/folders/1399
JBEI-099149	*ATCC 1015 pyrG− ΔglaA/P*_*glaA*_*-A5IL97*	This study.	https://registry.jbei.org/folders/1399
JBEI-099151	*ATCC 1015 pyrG− ΔmstC ΔglaA/ P*_*glaA*_*-A5IL97*	This study.	https://registry.jbei.org/folders/1399

### Plasmids

This study builds off of pre-existing Cas9 expression of the pFC332 shuttle plasmids for *A*. *niger* [22]. The plasmids express an *A*. *niger* codon optimized Cas9 under expression of the TEF-1 promoter. These contain the *A*. *nidulans* AMA1 replication cassette which mediates replication in multiple species of filamentous fungi [40]. The plasmid contains an hygromycin (*hph*) resistance marker for the selection of the plasmid. All plasmids were re-sequenced before proceeding further. Each transformation has been executed with a positive control, using two plasmids pFC330 (*pyrG* marker) and pFC332 (*hph* marker), and a negative control, using water.

### Construction of sgRNA

All of the sgRNA used, except for the *albA* sgRNA [22], were designed using the CRISPOR algorithm [41] and chosen to minimize off-target mismatches (Table 3). Once the sgRNA were chosen using the CRISPOR algorithm, they were prepared and tested *in vitro* using the Guide-it sgRNA Screening Kit (Takara). After the sgRNA were validated *in vitro*, they were amplified for transformation using the GeneART gRNA synthesis (Thermo Fisher). The concentration of sgRNA obtained after purification was ~10 μg/μL (Nanodrop). 20 μg sgRNA were used for each transformation to reach an optimal efficiency.

**Table 3:** Sequence of sgRNAs with original source.

Gene targeting	Sequencing	name	Source
*albA*	AGTGGGATCTCAAGAACTAC	sgRNA001	[22]
*glaA* 5'	CTGTGCAGACGAGGCCGCTC	sgRNA002	CRISPOR.tefor.net
*glaA* 3'	TCTACACGAAGGAAAGACCA	sgRNA003	CRISPOR.tefor.net
*mstC* 5'	TCCGCGTTGTATGAATCCAC	sgRNA004	CRISPOR.tefor.net
*mstC* 3'	GTGCCAGGCAGCCTGACCGG	sgRNA005	CRISPOR.tefor.net

### Donor DNA

#### DNA design

Each donor DNA (cDNA) contained the *pyrG* gene and was flanked with 1000 bp or 1500 bp HR arms for efficient integration [25].

#### DNA preparation

The preparation of the donor cDNA was performed via PCR cloning or purchased from Genscript (https://www.genscript.com/) (Table 4). The cDNA was integrated into the plasmid pUC57, transformed into DH10b competent cells (New England Biolabs, NEB) and selected on LB with 100 μg/mL carbenicillin plates. The resulting plasmids (Table 4) were sequence verified by Quintara (https://www.quintarabio.com/). The plasmids were used as the template to generate linear cDNAs by PCR amplification using Phusion Hot Start II (Thermo Fisher) and their respective primers (S1 Table). The four cDNAs PCR products were purified and concentrated to 1 μg/μL and 10 μg was used per transformation as described below.

**Table 4:** cDNA features and their accession information.

Strains	Plasmid	Amplicon	Gene target	Homology arms (bp)	Selectable marker	Sequence
JBEI-099138	pllk034	cDNA006	*albA*	1500	*pyrG*	https://registry.jbei.org/folders/1399
JBEI-099142	pllk036	cDNA008	*albA*	1000	*pyrG*	https://registry.jbei.org/folders/1399
JBEI-099144	pllk038	cDNA009	*glaA*	1000	*pyrG*	https://registry.jbei.org/folders/1399
JBEI-099146	pllk039	cDNA010	*mstC*	1000	*pyrG*	https://registry.jbei.org/folders/1399

### Transformation

Before transformation, *A*. *niger* was prepared for a protoplast-mediated transformation (PMT) [42], which consist of degrading the cell wall using VinoTaste Pro. After simultaneous transformation of Cas9, sgRNA, and the donor DNA into *A*. *niger pyrG−*, the mixture was incubated on ice for 20 minutes in a transformation solution (25% polyethylene glycol (6,000), 50 mM CaCl_2_, and 10 mM Tris HCl, pH 8.0). The mixture was plated on a 1% glucose minimal media containing agar and 1M sorbitol (MMA) + 300 μg/mL hygromycin, and the plates were incubated at 30°C. After transformation, the colonies were isolated on plates containing MMA + 300 μg/mL hygromycin. After visible growth but before the appearance of the first spores, the colonies were scooped out and isolated on slants containing only MMA. The Cas9 plasmid is lost in the absence of selective pressure (hygromycin). Once the colonies in the slants formed spores, the spores were isolated on plates containing MMA + 1.3 mg/mL 5-FOA + 1.2 mg/mL uracil. If the colonies were growing, they were re-isolated using MMA + 1.3 mg/mL 5-FOA + 1.2 mg/mL uracil plates again, then before the appearance of the first spores the colonies were scooped out and placed on slants containing MMA + 1.2 mg/mL uracil/uridine. For each transformation a minimum of 10 colonies were isolated, transformed on 5-FOA then re-isolated for analysis by PCR and sequencing (S6 Fig). To determine the efficacy of 5-FOA, the colonies were lysed and analyzed before and after exposure to 5-FOA. Note that if the *pyrG* marker needs to be recycled, it is recommended that the fungi recover between experiments. Also, manipulation of spores often leads to contamination and requires great care during the transformation [43]. The detail protocol “Transformation *Aspergillus niger* using Cas9, AMA1 vector, *pyrG* rescue marker and sgRNA *in vitro*” is available on protocols.io.

### Lysis

20 μL spores were harvested in 0.1% of tween buffer and mixed in 500 μL a solution containing 400 mM of Tris-HCl pH 8.0, 60 mM of ethylene diaminetetraacetic acid (EDTA) pH 8.0, 150 mM NaCl and 1% (v/v) sodium dodecyl sulfate (SDS). After incubation at room temperature for 10 minutes, 100 μL of a second solution containing 2 M potassium acetate, and 7.6% glacial acetic at pH 4.8 was added to the mixture. After centrifugation at 10,000 rpm, the supernatant containing the DNA was cleaned using isopropyl alcohol followed by 70% ethanol (EtOH). The ethanol was evaporated in a rotavapor (Vacufuge Plus Eppendorf) and the DNA was resuspended into 50 μL dH_2_O. The detail protocol “Lysis *Aspergillus niger*, extracting and purifying DNA” is available on protocols.io.

### PCR

Every transformation was analyzed by PCR (AB Applied Biosystems/Veriti 96 well Thermal Cycler) before 5-FOA and after 5-FOA (S1–S3, S4 and S5 Figs). We used LongAmp Taq DNA polymerase purchased from NEB and the primers synthesized by Integrated DNA Technology (IDT) (S2 and S3 Tables). The protocol followed was provided by NEB.

## Supporting information

S1 Fig Representative cDNA006 PCR before and after 5-FOA. (A) cDNA006 before 5-FOA, 5 colonies after transformation PCR amplification with 350/590, 3’125 bp. (B) After 5-FOA, 5 white colonies undergone *pyrG* excision, 1’386 bp, using both 1 kb Plus Ladder (Thermo Fisher/ 1kb Plus ready-to-use).
(DOCX)

S2 Fig 5-FOA plates of cDNA008 transformation. (A) First black colony after re-streaking on 5-FOA. (B) Second black colony after re-streaking on 5-FOA (C) First white colony after re-streaking on 5-FOA. (B) Second white colony after re-streaking on 5-FOA.
(DOCX)

S3 Fig Representative cDNA008 PCR before and after 5-FOA. (A) cDNA008 before 5-FOA, 5 colonies after transformation PCR amplification with 629/631, 3’434 bp. (B) After 5-FOA, 5 white colonies undergone pyrG excision, 695 bp. 1 kb Plus Ladder (Thermo Fisher/ 1kb Plus ready-to-use).
(DOCX)

S4 Fig Fig: Representative β-glucosidase (A5IL97) PCR and cDNA009 PCR after 5-FOA. (A) Amplification of the A5IL97 cassette of 5 colonies after transformation PCR with 608/609, 1’713 bp. (B) cDNA009 after 5-FOA of 5 colonies undergone pyrG excision, 719 bp. 1 kb Plus Ladder (Thermo Fisher/ 1kb Plus ready-to-use).
(DOCX)

S5 Fig Representative cDNA010 PCR before and after 5-FOA. (A) cDNA010 before 5-FOA, 5 colonies after transformation PCR amplification with 624/627, 3’753 bp. (B) After 5-FOA 5 colonies undergone pyrG excision, 1’024 bp. 1 kb Plus Ladder (Thermo Fisher/ 1kb Plus ready-to-use).
(DOCX)

S6 Fig Transformation. Schematic depiction of the process used for PMT transformation of *A*. *niger* using *pyrG* (-) auxotrophic marker.
(DOCX)

S1 Table Primers cDNA preparation. (DOCX)

S2 Table Primers B5FOA and A5FOA. Primers used for the amplification of amplicons before exposure of 5-FOA (B5FOA), after exposure of 5-FOA (A5FOA) and WT, to verify the length and the sequence (S5 Fig: Amplicons B5FOA and A5FOA).
(DOCX)

S3 Table Amplicons B5FOA and A5FOA. Amplification of amplicons before exposure of 5-FOA (B5FOA), after exposure of 5-FOA (A5FOA) and WT, to verify the length and the sequence.
(DOCX)
