# Supplementary material for: A new approach to Cas9-based genome editing in Aspergillus niger that is precise, efficient and selectable
Source: PLoS One. 2019 Jan 17;14(1):e0210243. doi: 10.1371/journal.pone.0210243 (PMC6336261; doi:10.1371/journal.pone.0210243)
Supplement: S2 Fig — (A) First black colony after re-streaking on 5-FOA. (B) Second black colony after re-streaking on 5-FOA (C) First white colony after re-streaking on 5-FOA. (B) Second white colony after re-streaking on 5-FOA. (DOCX) [file pone.0210243.s002.docx]

**S2 Fig: 5-FOAplates of cDNA008 transformation**

|  |
| --- |
| **S2 Fig:** **5-FOA plates of cDNA008 transformation.** (A) First black colony after re-streaking on 5-FOA. (B) Second black colony after re-streaking on 5-FOA (C) First white colony after re-streaking on 5-FOA. (B) Second white colony after re-streaking on 5-FOA. |
