# Supplementary material for: A new approach to Cas9-based genome editing in Aspergillus niger that is precise, efficient and selectable
Source: PLoS One. 2019 Jan 17;14(1):e0210243. doi: 10.1371/journal.pone.0210243 (PMC6336261; doi:10.1371/journal.pone.0210243)
Supplement: S4 Fig — (A) Amplification of the A5IL97 cassette of 5 colonies after transformation PCR with 608/609, 1’713 bp. (B) cDNA009 after 5-FOA of 5 colonies undergone pyrG excision, 719 bp. 1 kb Plus Ladder (Thermo Fisher/ 1kb Plus ready-to-use). (DOCX) [file pone.0210243.s004.docx]

**S4 Fig: Representative β-glucosidase (A5IL97) PCR and cDNA009 PCR after 5-FOA**

| 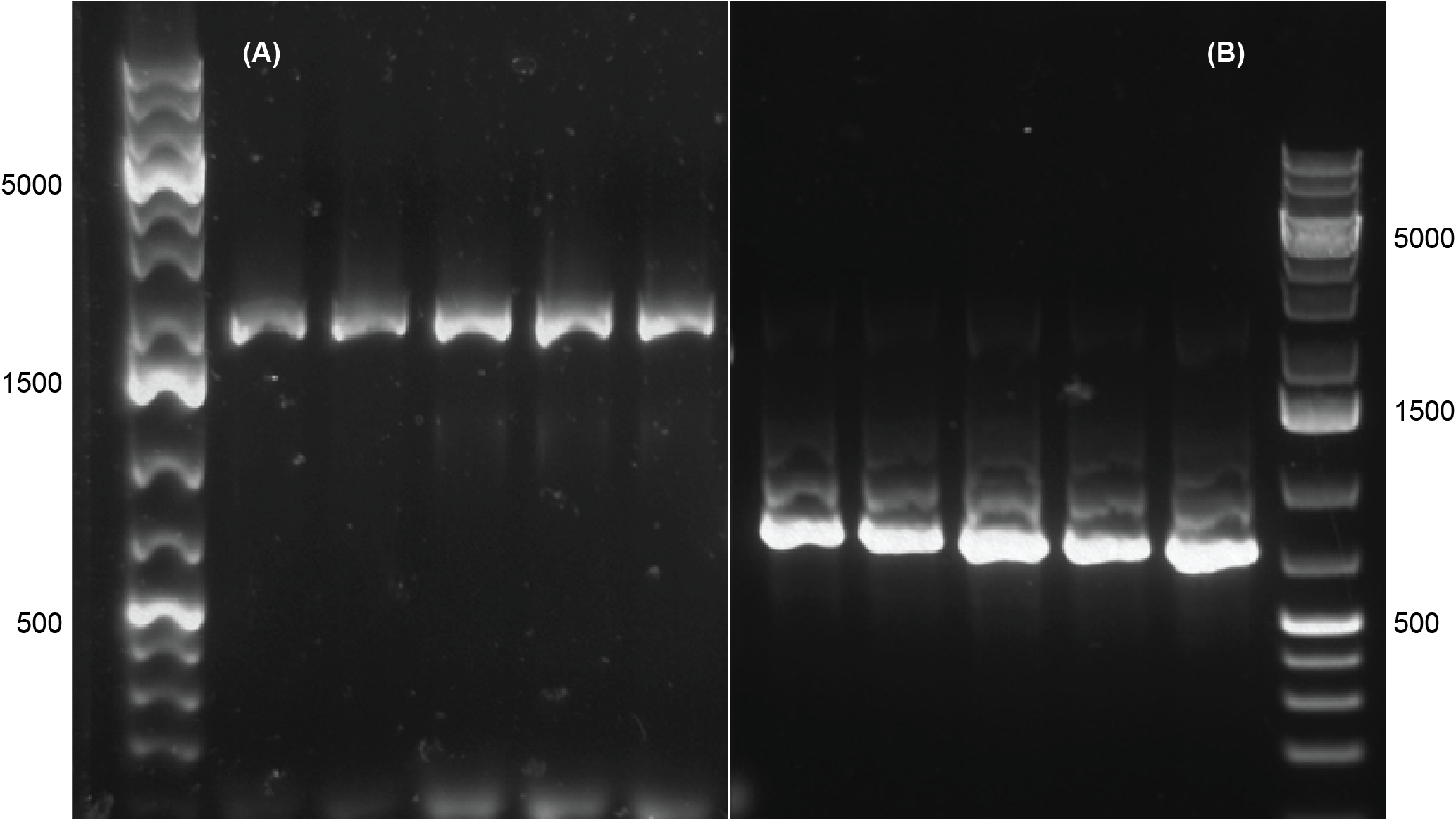 |
| --- |
| **S4 Fig: Representative β-glucosidase (A5IL97) PCR and cDNA009 PCR after 5-FOA** (A) Amplification of the A5IL97 cassette of 5 colonies after transformation PCR with 608/609, 1’713 bp. (B) cDNA009 after 5-FOA of 5 colonies undergone pyrG excision, 719 bp. 1 kb Plus Ladder (Thermo Fisher/ 1kb Plus ready-to-use). |
