# Supplementary material for: A new approach to Cas9-based genome editing in Aspergillus niger that is precise, efficient and selectable
Source: PLoS One. 2019 Jan 17;14(1):e0210243. doi: 10.1371/journal.pone.0210243 (PMC6336261; doi:10.1371/journal.pone.0210243)
Supplement: S5 Fig — (A) cDNA010 before 5-FOA, 5 colonies after transformation PCR amplification with 624/627, 3’753 bp. (B) After 5-FOA 5 colonies undergone pyrG excision, 1’024 bp. 1 kb Plus Ladder (Thermo Fisher/ 1kb Plus ready-to-use). (DOCX) [file pone.0210243.s005.docx]

**S5 Fig: Representative cDNA010 PCR before and after 5-FOA**

| 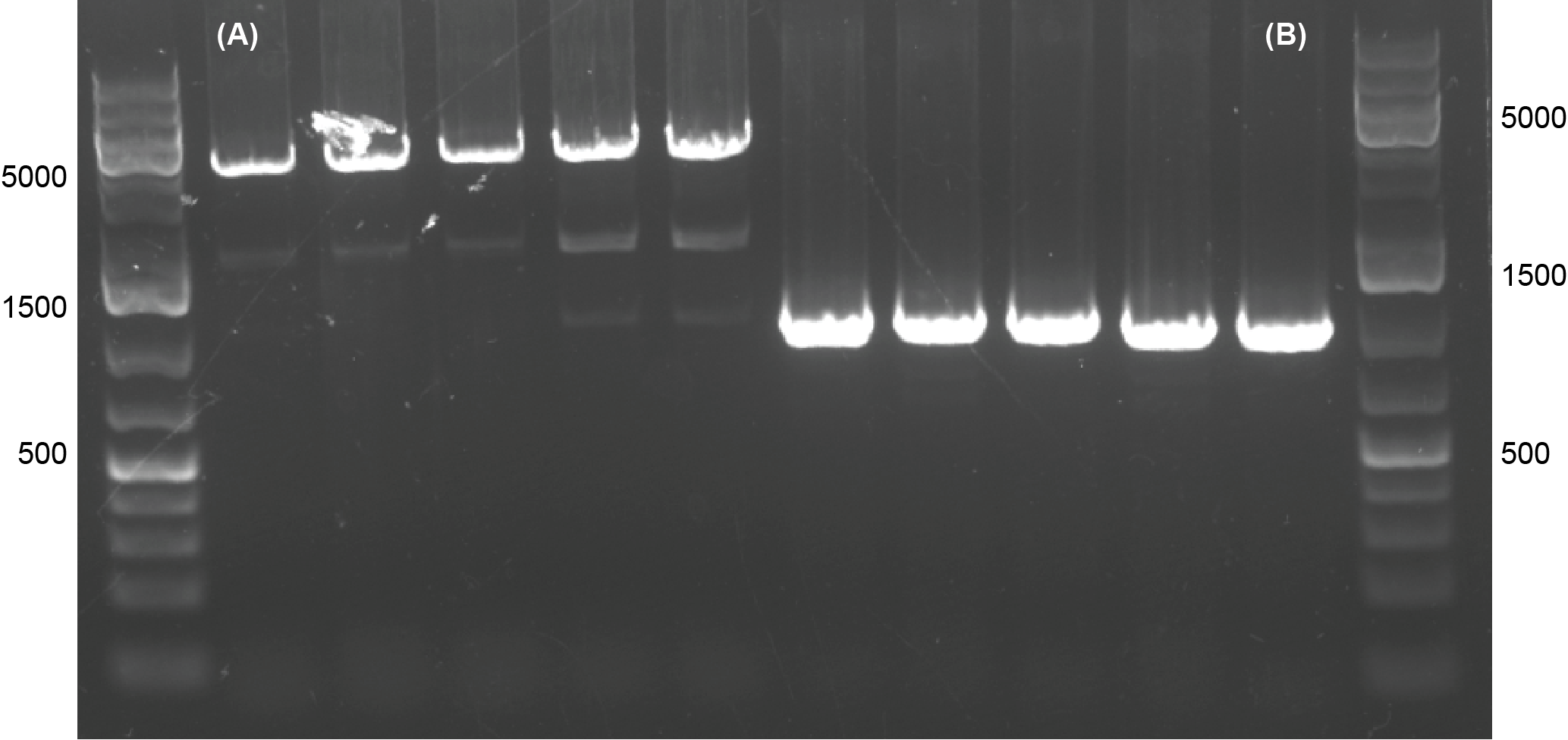 |
| --- |
| **S5 Fig: Representative cDNA010 PCR before and after 5-FOA.** (A) cDNA010 before 5-FOA, 5 colonies after transformation PCR amplification with 624/627, 3’753 bp. (B) After 5-FOA 5 colonies undergone pyrG excision, 1’024 bp. 1 kb Plus Ladder (Thermo Fisher/ 1kb Plus ready-to-use). |
