# Supplementary material for: A new approach to Cas9-based genome editing in Aspergillus niger that is precise, efficient and selectable
Source: PLoS One. 2019 Jan 17;14(1):e0210243. doi: 10.1371/journal.pone.0210243 (PMC6336261; doi:10.1371/journal.pone.0210243)
Supplement: S6 Fig — Schematic depiction of the process used for PMT transformation of A. niger using pyrG (-) auxotrophic marker. (DOCX) [file pone.0210243.s006.docx]

**S6 Fig: Transformation**

| 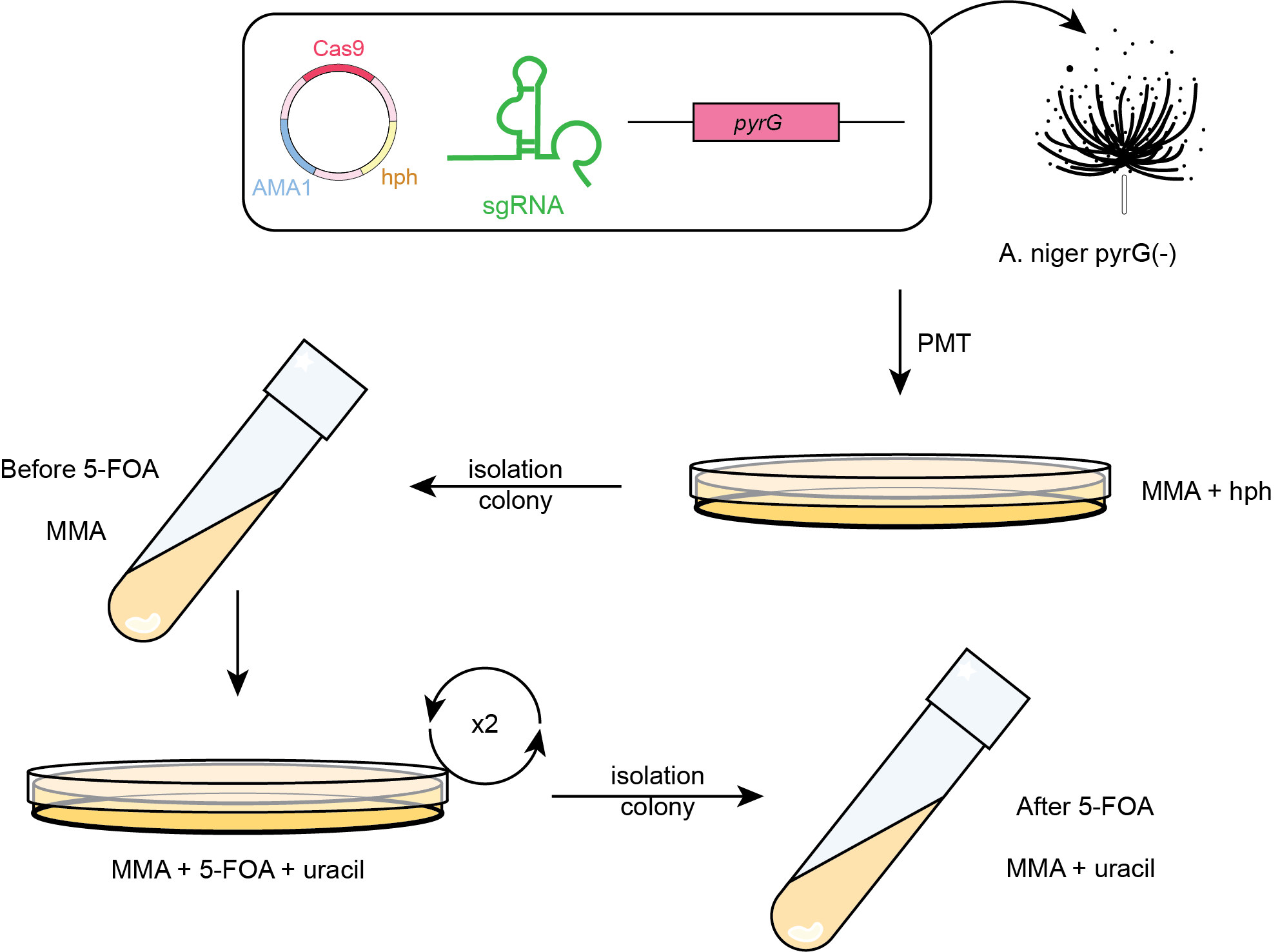 |
| --- |
| **S6 Fig:** **Transformation**. Schematic depiction of the process used for PMT transformation of *A. niger* using *pyrG* (-) auxotrophic marker. |
