# Supplementary material for: A new approach to Cas9-based genome editing in Aspergillus niger that is precise, efficient and selectable
Source: PLoS One. 2019 Jan 17;14(1):e0210243. doi: 10.1371/journal.pone.0210243 (PMC6336261; doi:10.1371/journal.pone.0210243)
Supplement: S1 Table — (DOCX) [file pone.0210243.s007.docx]

**S1 Table: Primers cDNA preparation**

| cDNA | Primers |  | Sequence | Length |
| --- | --- | --- | --- | --- |
| cDNA006 | LLK554 | Forward | CCGACAGACTTGGCGAAG | 4’758 bp |
|  | LLK555 | Reverse | CCTGGCTCATTGGGGCCAA |  |
| cDNA008 | LLK554 | Forward | CCGACAGACTTGGCGAAG | 4’258 bp |
|  | LLK582 | Reverse | AAACGTAGACATCACCAGCC |  |
| cDNA009 | LLK587 | Forward | CAAGTATATGATGCGGTAGTGGAATCT | 6’324 bp |
|  | LLK588 | Reverse | GGCTATGCATTGAATGACAGTG |  |
| cDNA010 | LLK595 | Forward | CGAAGAAGATTCCAGGAACG | 4’239 bp |
|  | LLK596 | Reverse | CACCAAGTAAGGTTCGTATAT |  |
