# Supplementary material for: A new approach to Cas9-based genome editing in Aspergillus niger that is precise, efficient and selectable
Source: PLoS One. 2019 Jan 17;14(1):e0210243. doi: 10.1371/journal.pone.0210243 (PMC6336261; doi:10.1371/journal.pone.0210243)
Supplement: S2 Table — Primers used for the amplification of amplicons before exposure of 5-FOA (B5FOA), after exposure of 5-FOA (A5FOA) and WT, to verify the length and the sequence (S5 Fig: Amplicons B5FOA and A5FOA). (DOCX) [file pone.0210243.s008.docx]

**S2 Table: Primers B5FOA and A5FOA**

Primers used for the amplification of amplicons before exposure of 5-FOA (B5FOA), after exposure of 5-FOA (A5FOA) and WT, to verify the length and the sequence (Supplemental Figures 5: Amplicons B5FOA and A5FOA).

| Primers Name | Sequence |
| --- | --- |
| 350 | ATGCGAAGCTGACCAATGACAAGAC |
| 589 | ATGAAGATGGAGGATTCGAG |
| 590 | ACTGTGACACCAATGATTAG |
| 603 | CTCTTCTTTACTCTGATAGCTTGAC |
| 604 | GATCAGACCAGTAGGTAAACTAG |
| 608 | AGTTTCCAGAAGGGTTTTTGTG |
| 609 | CAAGCTATCAGAGTAAAGAAGAGG |
| 624 | ATTCAGGTACTCACCGTCCACATC |
| 627 | TCTTCTAAGTTCCTGCTCACTCTCC |
| 629 | GCGAAGAAGATACCTGGAAGGTC |
