# Supplementary material for: A new approach to Cas9-based genome editing in Aspergillus niger that is precise, efficient and selectable
Source: PLoS One. 2019 Jan 17;14(1):e0210243. doi: 10.1371/journal.pone.0210243 (PMC6336261; doi:10.1371/journal.pone.0210243)
Supplement: S3 Table — Amplification of amplicons before exposure of 5-FOA (B5FOA), after exposure of 5-FOA (A5FOA) and WT, to verify the length and the sequence. (DOCX) [file pone.0210243.s009.docx]

**S3 Table: Amplicons B5FOA and A5FOA**

Amplification of amplicons before exposure of 5-FOA (B5FOA), after exposure of 5-FOA (A5FOA) and WT, to verify the length and the sequence.

| construct – strain A. niger | Primers | ^T[C]^ | B-5FOA | A-5FOA | WT |
| --- | --- | --- | --- | --- | --- |
| *cDNA006 – albA (-)* | 350/590 | 55 | 3’125 bp | 1'386 bp | 1’367 bp |
| *cDNA008 – albA (-)* | 629/631 | 55 | 3’434 bp | 695 bp | 1’695 bp |
| *cDNA009 – ΔglaA/PglaA-A5IL97* | 603/604 | 54 | 3’458 bp | 719 bp | 0 bp |
| *cDNA010 - ΔmstC* | 624/627 | 58 | 3’753 bp | 1’024 bp | 3’213 bp |
| *cDNA009****_010 ΔmstC****/ ΔglaA/PglaA-A5IL97* | 624/627 | 58 | 3’753 bp | 1’024 bp | 3’213 bp |
| *β -glucosidase-A5IL97* | 608/609 | 55 | 1’713 bp | 1’713 bp | 0 bp |
